# Dr. Charles Bell Taylor

**Published:** 1909-04-24

**Authors:** 


### OBITUARY.

Dr. Charles Bell Taylor, the eminent ophthalmic surgeon, died at Nottingham, on April 14 after a very short illness at the age of 80. Dr. Taylor received his medical education at the University of Edinburgh and in Paris. He obtained the M.R.C.S.Eng. in 1852, and, after acting as medical superintendent of the Walton Lodge Lunatic Asylum at Liverpool, he took the M.D. degree at Edinburgh in 1854, and shortly afterwards settled in Nottingham to practise. In 1859 a vacancy for a surgeon to the new Eye Infirmary at Nottingham turned his attention towards ophthalmic surgery, in which branch of the profession he showed increasing skill as years went by. His reputation as a successful operator on the eye soon travelled far beyond the neighbourhood, and his opinion was valued by a very wide circle of practitioners. In spite of his advanced age, he continued to see patients, and quite lately advised the operation for cataract upon "General" Booth, which has since been successfully performed. His most important published work was "Clinical Lectures on Diseases of the Eye."

